# The development of nurse prescribing in mental health services: Outcomes from five national surveys 2004–2019

**DOI:** 10.1111/jonm.13588

**Published:** 2022-03-21

**Authors:** Neil Brimblecombe, David Dobel‐Ober

**Affiliations:** ^1^ Institute of Health and Social Care London South Bank University London UK; ^2^ Research and Innovations Midlands Partnership NHS Foundation Trust Stafford UK

**Keywords:** advanced practice, mental health services, non‐medical prescribing, psychiatric/mental health nursing

## Abstract

**Aim:**

This study aimed to explore data from national surveys of nurse prescribing in England's National Health Service mental health services.

**Background:**

Nurse prescribing is increasing worldwide. Reports describing long‐term developments after implementation are rare.

**Methods:**

Five surveys were distributed to all mental health organisations between 2004 and 2019.

**Results:**

Response rates increased from 54% (*n* = 45/83) in 2004 to 79% (*n* = 42/53) in 2019. The estimated proportion of mental health nurses who were prescribers increased to 4.3% by 2019. Distribution between clinical practice areas did not change significantly over time, with the largest numbers in community mental health teams. The proportion of nurse prescribers actively prescribing increased from 76.4% in 2014 to 87.8% in 2019. Independent prescribing became the predominant approach, with supplementary prescribing rarely used as the sole model within organisations. The scale of implementation varied markedly between organisations.

**Conclusions:**

Although nurse prescribing in mental health services has grown over time, growth has slowed and is variable at local level.

**Implications for Nursing Management:**

Organisations considering the introduction or growth of nurse prescribing should note the evidenced preference for an independent prescribing model to date and consider how to avoid unwarranted variation in nurse prescriber role distribution.

## 1 BACKGROUND

Non‐medical prescribing (NMP) typically aims to increase patient access to medicines and make better use of the skills of health professionals, whilst not compromising patient safety (Cope et al., 2016). The number of countries with laws permitting some form of NMP has steadily grown over the last 20 years, with nursing being the profession most often granted such powers. Nurse prescribing is an advanced clinical skill and a required component of most advanced clinical practice (ACP) roles in nursing (Fong et al., 2015). The International Council of Nurses (n.d.) estimate that 70 countries have ACP nursing roles currently or are planning to do so. Maier (2019) identified 13 European countries with nurse prescribing powers, 10 introducing this since 2010. Despite frequent opposition from medical lobbies (Day, 2005; Elsom et al., 2009; Lim et al., 2017; Zarzeka et al., 2019), nurse prescribing seems likely to continue to spread, with more countries debating its introduction (Badnapurkar et al., 2018; Ling et al., 2018).

The development of nurse prescribing has taken place in a context of international workforce shortages (Kakuma et al., 2011; Lancet, 2018), large treatment gaps, particularly in lower and middle‐income countries (World Health Organization, 2011), growth of neo‐liberal and managerialist agendas within health care systems (Hewko & Cummings, 2016) and international evidence that health professionals often perform tasks for which they are overqualified (OECD, 2013).

Systematic reviews have compared the effectiveness of nurse prescribing and medical prescribing. A Cochrane review (Weeks et al., 2016) identified 26 studies and concluded that nurse prescribers practising in a variety of settings can achieve outcomes in the management of chronic disease and preventive health care comparable with those with medical prescribers. Noblet et al. (2018) concluded that the limited evidence available from randomized control trials suggests that nurse prescribing is safe and can provide beneficial clinical outcomes, although did not comment on generalizability of this judgement to specific clinical specialities, such as mental health, with no trials having examined nurse prescribing in general mental health services in the United Kingdom to date.

### 1.1 Nurse prescribing internationally

Internationally, data regarding distribution and characteristics of NPs in mental health services are mostly reported within wider studies of nurse prescribing across all specialities. In the United States, surveys have taken place of advanced nurse practitioners (ANPs) with prescribing powers. In 2019, 4.2% of ANPs were reported as working predominantly in mental health settings (American Association of Nurse Practitioners [AANP], 2019), with such roles being more numerous in areas with low numbers of psychiatrists (Feldman et al., 2003; Goolsby, 2011). In Australia, surveys have indicated a low proportion of mental health‐focused nurse prescribers (Buckley et al., 2013; Fong et al., 2020), whereas in New Zealand, a study identified less than 1% of patients receiving nurse prescribing were mental health/substance misuse service users (Poot et al., 2017).

In the United Kingdom, cross‐sectional studies have provided some specific information regarding distribution of nurse prescribers in mental health services, in addition to the five surveys reported herein. A national survey of nurse prescribers in Wales identified only 8 mental health nurses (MHNs) from 321 respondents (Courtenay et al., 2017), whereas Latter et al. (2011) reported that 5.5% of mental health inpatient wards/units and 47.7% of community mental health units in England had a qualified nurse prescriber. A survey of substance misuse services in England and Scotland identified 324 nurse prescribers, some of whom were employed outside the National Health Service (NHS) (Mundt‐Leach & Hill, 2014). In relation to advanced practice type roles, a national survey of MHN consultants in England identified 35.7% of respondents as NPs (Brimblecombe et al., 2019).

### 1.2 Scope of nurse prescribing

There is significant variation in the scope of prescribing authority between countries (Maier, 2019). The United Kingdom (including England) has broader nurse prescribing powers than most other countries (National Institute for Health and Care Excellence, 2019). Supplementary prescribing has been open to MHNs since 2003, being a voluntary partnership between an independent prescriber (commonly a medical practitioner) and the supplementary prescriber, to implement an agreed patient‐specific clinical management plan with the patient's agreement. Independent prescribing has also been available for MHNs since 2006, whereby a nurse prescriber can prescribe any medicine (excepting certain controlled drugs), provided they are competent to do so.

The Nursing and Midwifery Council (NMC) regulates the UK nursing profession and records nurse prescribing qualifications. Registered nurses can qualify as prescribers following completion of an additional postgraduate level training programme. The NMC is unable to provide specific data regarding the number of prescriber qualified MHNs (NMC, personal communication, 2019). However, overall numbers of nurses from all specialities with the full prescribing qualification are available for time periods close to that of the surveys described herein (NMC, personal communication, 30/10/2019).

This paper reports and analyses selected data from five surveys of nurse prescribing in mental health services in England, in 2004 (Gray et al., 2005), 2005, 2008 (Dobel‐Ober et al., 2010), 2014 (Dobel‐Ober & Brimblecombe, 2016) and 2019. Author 1 was involved with all five surveys and Author 2 with the latter three.

## 2 AIM

This study aimed to describe the development of nurse prescribing in NHS mental health services in England, including changes in distribution and type.

## 3 METHOD

For each survey, an invitation to participate was sent to the director of nursing of every NHS trust that then provided mental health services. The invitation to participate explained the purpose of the survey and provided a summary of previous survey outcomes.

Directors of nursing were chosen as contacts, as having good awareness of local workforce developments and high degree of influence over nurse prescribing implementation.

Follow‐up to non‐responders was by email after 3 months and, in the 2014 and 2019 surveys, further contact by phone.

### 3.1 The questionnaire

The questionnaire was originally designed in 2003 by the National Institute for Mental Health in England Non‐Medical Prescribing Advisory Group, in the context of a lack of national data regarding nurse prescribing in mental health services.

The surveys were not originally planned as a time series. Subsequent surveys were largely based on the 2003 questionnaire but were amended to reflect changes in the legal prescribing framework and the structure of mental health services. In 2014 and 2019, additional questions were included regarding governance and planning activities, and a free‐text question as to perceived barriers to implementation.

### 3.2 Analysis

Quantitative survey data were analysed with SPSS 25 (IBM). Open‐ended questions were analysed by thematic analysis (Smith, 2000). In all surveys, respondents were asked to report the number of MHNs employed by their organisation, but few provided this information, and those responses are not reported here. Average numbers of MHNs in responding trusts were extrapolated as a proportion of the total MHN workforce in England, based on NHS workforce statistics. Those figures were then used to estimate the proportional change in nurse prescriber numbers in mental health services. This process of mean imputation may underestimate variation (Eekhout et al., 2012).

### 3.3 Ethical issues

Surveys in 2004 and 2005 were carried out as a workforce data gathering exercise for a national body (National Institute for Mental Health). Later surveys were registered as service evaluations with the R&D department of the NHS trust of Author 2, as the projects did not meet the criteria for research following the Health Research Authority (HRA) guidelines (HRA, n.d.). The survey did not record personal identifiers, and individual trust's data were anonymised.

## 4 RESULTS

Response rates rose over time, in a context of NHS trusts increasing in size but reducing in number through this period: 2004, 54% (45/83); 2005, 66% (53/83); 2008, 59% (39/56); 2014, 75% (39/52); and 2019, 79% (42/53). The estimated proportion of all NHS MHNs (NHS Digital, 2020) who were prescribers increased from 0.5% in 2004 to 4.3% in 2019.

**FIGURE 1:**
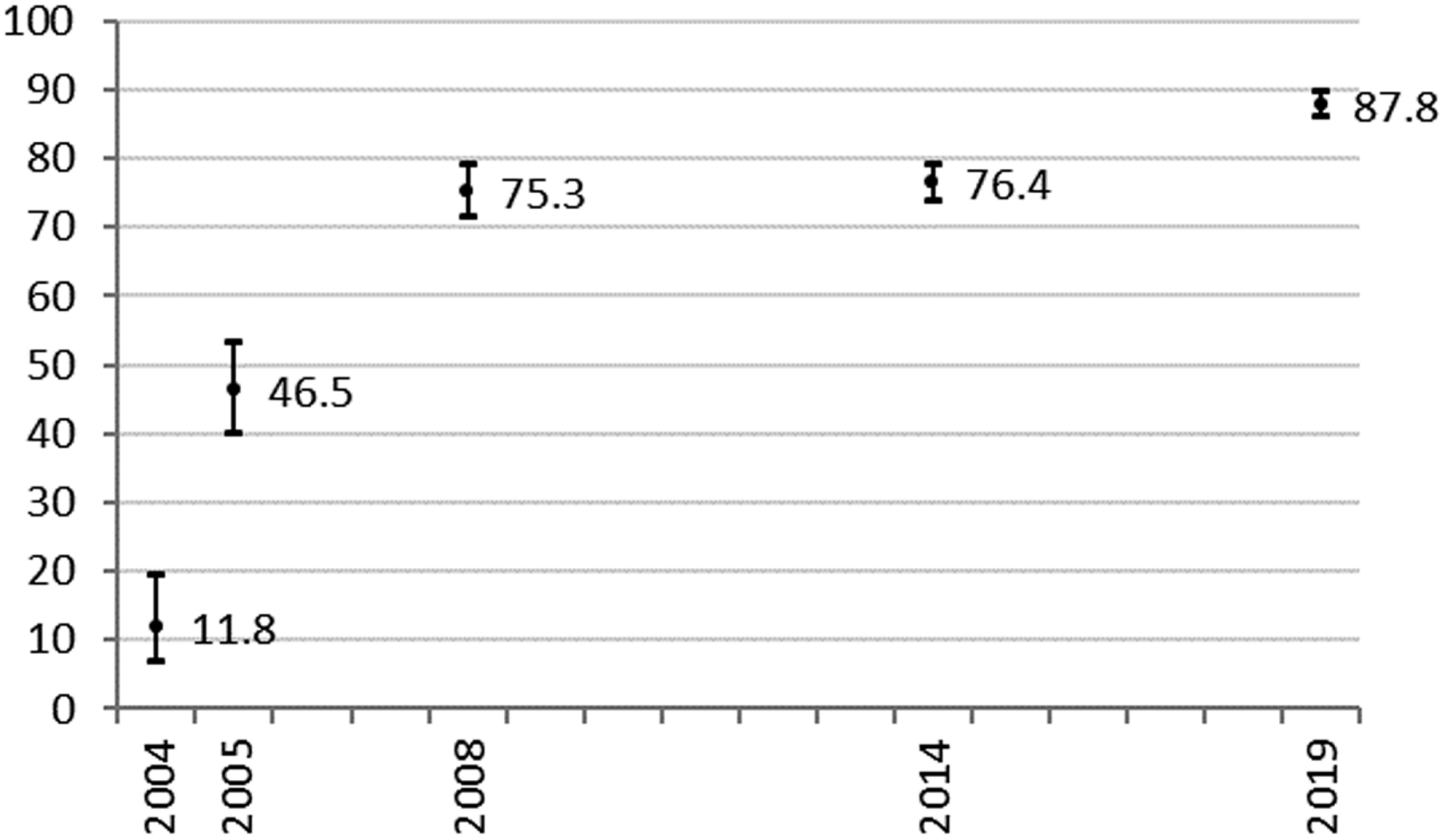
Proportion of qualified non‐medical prescribers perceived as active prescribers (with 95% confidence intervals)

**FIGURE 2:**
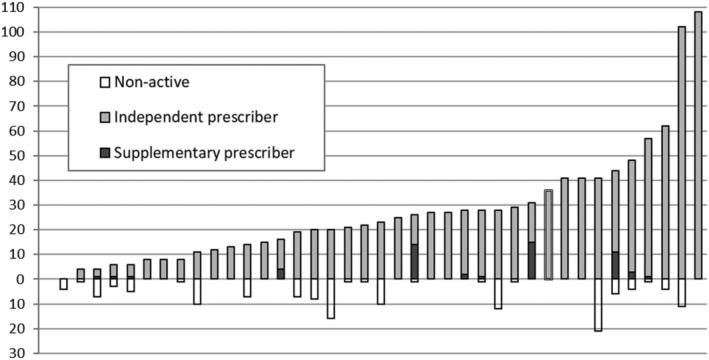
Distribution of nurse prescribers and type of prescribing, by individual trust (2019)

The mean number of nurse prescribers in participating trusts increased from 2004 (*n* = 2.3) to 2014 (*n* = 35.0) and then fell from 2014 to 2019 (*n* = 29.2). However, the mean number described as active (currently prescribing) reduced only marginally, from 26.9 to 26.3 in this latter period. In 2019, 16 trusts reported that all their nurse prescribers were active prescribers. This compared to 11 in 2014 and 15 in 2008 (figures not available for 2004 and 2005). The overall proportion of nurse prescribers described as active increased significantly between 2005 (46.5%, 95% confidence interval [CI] [39.9, 46.5]) and 2008 (75.3% [71.3, 78.9]) and between 2014 (76.4% [73.7, 79.0]) and 2019 (87.8% [85.8, 89.6]) (Figure 1). Successive surveys revealed large variations in the number of nurse prescribers employed between trusts; this was still apparent in the latest iteration in 2019 (Figure 2).

Overall numbers of all nurses with the full nurse prescriber qualification across the United Kingdom were available for time periods close to that of the surveys described herein (NMC, personal communication, 30/10/2019). Total numbers increased from 4199 in 2004/2005 to 42,273 in 2018/2019. This represents an overall increase of 1007% over the 15‐year period. The estimated number of MHN prescribers increased from 191 to 1548 over the same period (Table 1), an increase of 810%.

**TABLE 1:** Distribution of mental health nurse and mental health nurse prescribers

Survey year	2004	2005	2008	2014	2019
Trusts providing mental health services in England	83	80	66	52	53
Number of participating trusts (response rate)	45 (54%)	53 (66%)	39 (59%)	39 (75%)	42 (79%)
Mean NPs per participating trust	2.3	4.6	15.9	35.0	29.2
Mean MHNs per trust (estimated from NHS census)	476	506	615	722	683
% MHNs qualified as NPs (estimated from NHS census)	0.5%	0.9%	2.5%	4.0%	4.3%
Mean NPs per trust currently prescribing	0.3	1.9	11.8	26.9	26.3
Mean NPs in training per trust	2.8	2.4	3.1	3.7	4.6
% MHNs training in NP (estimated from NHS census)	0.6%	0.5%	0.5%	0.5%	0.7%
MHN total (England)	39,536	40,457	40,602	37,553	36,183
NPs total (England, estimated)	191	368	1049	1820	1548

Abbreviations: MHNs, mental health nurses; NPs, nurse practitioners.

### 4.1 Type of prescribing

**FIGURE 3:**
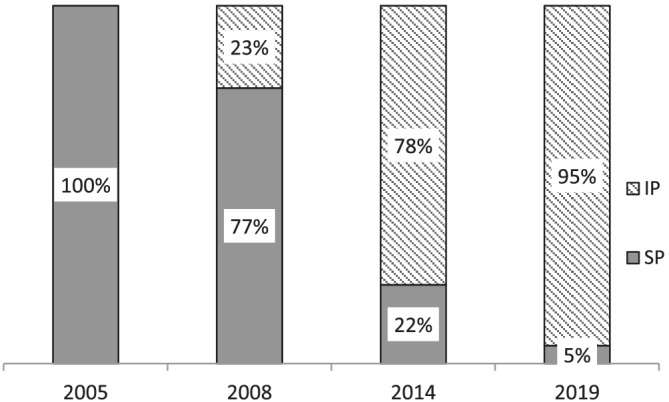
Proportions of independent and supplementary only nurse prescribers. IP, independent prescriber; SP, supplementary prescriber

Figure 3 illustrates distribution of type of prescribing practice in four surveys. In 2004 and 2005, supplementary prescribing was the only approach open to nurses. The proportion of nurse prescribers who practised independent prescribing increased from 23% (*n* = 98) in 2008 to 95% (*n* = 975) in 2019. The proportion of trusts with either formal (with a written policy) or informal (with no written policy) process to progress nurse prescribers from only supplementary prescribing to independent prescribing (with or without supplementary prescribing as well) reduced between 2008 (*n* = 20, 58%) and 2019 (*n* = 18, 47%). In 2019, 9 trusts indicated that all their nurse prescribers were independent, whereas 22 trusts (52%) reported the use of either a formal or informal process to make the transition for individual nurse prescribers from supplementary to independent prescribing.

### 4.2 Clinical area of practice

Nurse prescribers were reported as working in diverse clinical areas in surveys from 2005 to 2019 (data were not sought in 2004). Community mental health teams were consistently the clinical practice area with most nurse prescribers. Changes in the proportion of nurse prescribers working in each clinical area did not reach levels of significance between the four surveys (*p* > .05) (see Table 2).

**TABLE 2:** Active nurse prescribers by service setting 2005, 2008, 2014 and 2019 (data not available for 2004)

	2005			2008			2014			2019		
	*n*	%	CI	*n*	%	CI	*n*	%	CI	*n*	%	CI
CMHT	61	29	[37.89, 20.11]	97	27	[35.7, 18.3]	243	30	[38.98, 21.02]	280	26	[34.6, 17.4]
Older people community	42	20	[27.84, 12.16]	53	15	[22, 8]	113	14	[20.8, 7.2]	166	15	[22, 8]
Drugs and alcohol	29	14	[20.8, 7.2]	67	19	[26.69, 11.31]	163	20	[27.84, 12.16]	163	15	[22, 8]
Assertive outreach teams	18	8	[13.32, 2.68]	25	7	[12, 2]	44	5	[9.27, 0.73]	33	3	[6.34, −0.34]
Crisis/home treatment	12	6	[10.65, 1.35]	26	7	[12, 2]	66	8	[13.32, 2.68]	105	10	[15.88, 4.12]
Acute inpatient	8	4	[7.84, 0.16]	17	5	[9.27, 0.73]	31	4	[7.84, 0.16]	69	6	[10.65, 1.35]
Older people inpatient	8	4	[7.84, 0.16]	12	3	[6.34, −0.34]	30	4	[7.84, 0.16]	22	2	[4.74, −0.74]
Other	35	16	[23.19, 8.81]	59	17	[24.36, 9.64]	127	16	[23.19, 8.81]	252	23	[31.25, 14.75]

Abbreviations: CI, confidence interval; CMHT, community mental health team.

### 4.3 Barriers and facilitators to implementation

In 2014 and 2019, a free‐text question asked about barriers to implementation. In 2014, 16 trusts identified 35 barriers, and in 2019, 17 trusts identified 26 barriers. The most common issue in both surveys related to medical support (2014, *n* = 8) (2019, *n* = 7), examples including ‘opposition and poor co‐operation from consultants’ and ‘availability of medical supervision’. In 2019, there were four reports of issues with the universities providing nurse prescriber training, for example, difficult entry criteria for students, but none in 2014. The lack of a strategic approach towards implementing nurse prescribing was cited as a barrier more frequently in 2014 (*n* = 6) than in 2019 (*n* = 2). Funding issues for training were identified more in 2019 (*n* = 4) than in 2014 (*n* = 1).

The 2019 survey confirmed 2014 findings that all responding trusts with nurse prescribers had a named person in a lead role to support nurse prescribing practice, although the amount of time available to these roles was often very small, with 66% of leads dedicating less than 1 day a week to the role (63% in 2014).

## 5 DISCUSSION

The five surveys provide a unique description of nurse prescribing in a major clinical speciality of a large national health care system over an extended period. To the authors' knowledge, detailed longitudinal quantitative surveys of nurse prescribing have only been reported elsewhere through surveys of ANPs in the United States, for instance, Goolsby (2011). The findings from the surveys described here will allow for comparison with developments in other specialities and/or countries. This is timely, with the continuing expansion of nurse prescribing internationally.

The survey response rate generally increased over time, from 54% in 2004 to 79% in 2019. These response rates are higher than average for organisational surveys (Baruch & Holtom, 2008). The authors hypothesize that the increase in response rates may reflect the overall increase in use of nurse prescribing in health care organisations, with fewer trusts having low numbers of nurse prescribers or little governance in place. Trusts with little nurse prescribing activity may be more reluctant than others to participate in surveys, even where data are anonymised for publication.

### 5.1 Distribution

The rapid increase rate in nurse prescriber numbers did not persist to 2019, when the total number of nurse prescribers was lower than in 2014, although numbers of nurse prescribers reported as actively prescribing remained similar. Although trainee nurse prescriber numbers increased in each survey, existing nurse prescribers moving into non‐prescribing roles and retiring may explain the reduction in numbers from 2014 to 2019.

When compared with the growth rate of nurse prescribers from all specialities as recorded by the NMC, mental health growth is slower, although NMC figures may exaggerate the number of nurse prescribers in practice, not accounting for those who cease to use their prescribing qualifications but who remain registered as a nurse. Most notably, between 2014/2015 and 2019, overall numbers of NMC registered prescribers increased by 38% (NMC, personal communication, 2020), compared with a small decrease in MHN prescribers identified from the 2014 and 2019 studies reported herein. No evidence is yet available as to why. With 86% of people receiving mental health treatment being prescribed medication (Lubian et al., 2016), a slower rate of growth in the speciality is worthy of further investigation.

The proportion of nurse prescribers who were reported as not actively prescribing reduced from 23.6% to 11.4% between 2014 and 2019. This may have resulted from the removal of prescriber status from those qualified prescribers who were not prescribing due to greater oversight within trusts and/or have reflected greater understanding of and support for the role. Courtenay et al. (2017), in a survey of nurse prescribers from all specialities, reported the main reason given for not prescribing was a change in individuals' roles. The level of non‐prescribing in the 2014 and 2019 surveys approximates that of 14% reported by Latter et al. (2011) in an English national survey of all specialities, suggesting that mental health results are not an outlier.

There was marked variation in the distribution of nurse prescribers between trusts. A systematic review of policy papers has concluded that across the NHS, generally, nurse prescribing appears embedded into practice (Graham‐Clarke et al., 2019), although variation in distribution is reported across the United Kingdom and between specialisms (Courtenay et al., 2011). Relatively slow development and wide variation in distribution of new roles such as ACPs and nurse consultants have also been noted in mental health services (Brimblecombe & Nolan, 2021; Brimblecombe et al., 2019). Greenhalgh et al. (2004) cite the extent to which a health care innovation becomes integrated into usual practice as depending on the interaction between features of the innovation, the adopters and the context. Innovations that have a demonstrable advantage in either effectiveness or cost‐effectiveness are more easily implemented. However, currently, there is no specific and robust evidence base for nurse prescribing in mental health services to support implementation, nor evidence as to any comparative outcomes in different mental health clinical settings. In this context, introduction of innovations, such as new roles, may be more likely to be influenced by attitudes of individual organisational leads (Brimblecombe et al., 2019). Adequately powered randomized control trials evaluating clinical and cost‐effectiveness are still required to evaluate NMP across clinical specialties, professions and settings (Noblet et al., 2018). Courtenay et al. (2011) comment that nurse prescribing roles are more likely to become embedded where a strategic approach to development is adopted by trusts. The data from the surveys herein imply that the leadership in different mental health trusts may perceive the value of nurse prescribing differently or that unknown local factors produce different workforce requirements.

The range of clinical areas where nurse prescribers practise is broad, although there is consistency as to where the largest number is found, that is, community mental health teams (26.5% of prescribers in 2008/2019). There has been no significant change in the proportion of nurse prescribers working in other areas over time. The question of why nurse prescribing is established in certain clinical areas was not addressed by this study but is important to understand.

### 5.2 Models of nurse prescribing and area of clinical practice

In 2004 and 2005, the only form of nurse prescribing available to mental health services was supplementary prescribing. This had only been introduced in 2003, leaving little time for trusts to identify areas that might benefit from its implementation, identify suitable candidates for the role and develop suitable governance systems. The evidence of the growing predominance of independent, rather than supplementary, prescribing models in the later surveys strongly suggests that that supplementary prescribing is not the favoured model in mental health services, except in some cases as part of a transitional pathway towards independent prescribing (Dobel‐Ober et al., 2013). A similar pattern has been identified in non‐mental health services (Courtenay & Carey, 2008). Independent prescribing allows greater flexibility and responsiveness in services, for example, in extended hours community teams, where medical cover is often limited. The evidence reporting differential frequency of use between the two prescribing models has implications for other countries. Narrower nurse prescribing powers than those available in England are more typical internationally. However, the above evidence suggests that a restrictive prescribing model, such as supplementary prescribing, is less likely to be the model preferred by clinical services than a less restrictive model, that is, independent prescribing.

There is no evidence available as to the extent of nurse prescribing by advanced clinical practitioners in England's mental health services to date in the studies reported here or elsewhere, excepting that by nurse consultants, who have some similar educational and role requirements (Brimblecombe et al., 2019). The development of ACP roles appeared slow initially in England's mental health services (Brimblecombe & Nolan, 2020); however, national encouragement for developing these roles (NHS England [NHSE], 2019) seems likely to ultimately increase nurse prescribing by default, as prescribing training is part of ACP training requirements. In countries where the route for introduction of nurse prescribing is solely via ACPs, then any spread of ACP roles will create growth in nurse prescribing, but ultimately, this growth may be less than if other routes to prescribing practice were also available.

### 5.3 Limitations

The lower levels of response in the earlier surveys and changes in the number and boundaries of trusts somewhat limit confidence as to the ability to report change over time. The lack of central recording of the field of practice by the NMC of qualified non‐medical prescribers made comparison of trends within mental health services compared with other specialties difficult, with only tentative conclusions being drawn from the sample in the surveys.

Some questions in the survey required a degree of interpretation by the participants, for example, as to the number of nurse prescribers who were not active. There is no national definition of ‘active’, and relatively few trusts reported have a formal policy in this regard. Answers may therefore have been calculated in somewhat different ways.

The authors consider it likely that trusts with particularly low numbers of nurse prescribers were less likely to respond to the survey. This would suggest that results may overestimate nurse prescriber numbers as a proportion of MHNs nationally, although later surveys would be more accurate in this regard.

Although data as to the prescribing practices of nurse prescribers have been usefully used in other surveys, this was not feasible for the studies herein due to the lack of a single national database encompassing the varied settings in which MHN prescribers practise.

NMP in professions other than nursing is an important issue but was not reported in this paper, as it was not explored in detail in the surveys.

## 6 CONCLUSIONS

This paper provides a unique insight into the development of nurse prescribing over a 15‐year period within a major specialty of a large‐scale national health service. The findings illustrate the importance of the type of nurse prescribing adopted, the challenge of variation in approach between local organisations and the likely implications of lacking a specialty‐specific evidence base.

The move away from the use of supplementary prescribing suggests that this approach is largely used as a developmental stage for new nurse prescribers before progressing to independent prescribing status and as such is not recognized as a significant contribution to service delivery in itself. This active choice by services provides countries considering adopting nurse prescribing a useful case study of organisational choice regarding preferred type of nurse prescribing, at least in mental health services.

The surveys suggest that where nurse prescribing is introduced, there will be variable take‐up of the role and variability in how it is utilized. The role of senior ‘champions’ within organisations may play a major role in the level of uptake, as may the attitude of senior medical staff.

Non‐prescribing once qualified can be a serious waste of resource and is found in these and other surveys, although here non‐prescribing reduced over a period of years. There is reported variation in this regard between countries, so high levels of non‐use may not be inevitable.

## 7 IMPLICATIONS FOR NURSING MANAGEMENT

Nurse prescribing is a skill set that potentially allows for service and workforce redesign and enhanced nursing employment pathways. The surveys reported in this paper illustrate that choices made by local organisation management are likely to have a large effect on how and to what extent nurse prescribing is utilized in services, especially in the absence of a robust, speciality‐specific evidence base. Managers across multiple organisations will need to consider what level of variation between organisations is justifiable. The lack of change over time as to which clinical areas have most nurse prescribers suggests that managers may need to ensure processes of ongoing evaluation take place to ascertain whether distribution is the most effective possible.

Although relatively rare, some challenges were identified regarding support from medical staff for nurse prescribing roles, which nurse managers may need to proactively address.

The results clearly indicate that independent prescribing is the model of prescribing most used in mental health services. Therefore, nurse managers, where nurse prescribing is being considered for introduction, may consider prioritizing the development of independent prescribing where legally permissible and note that any national plans to introduce a form of nurse prescribing with less scope, for example, a supplementary prescribing model, may be perceived as less able to meet service needs.

## CONFLICT OF INTEREST

The authors declare no conflicts of interest.

## AUTHOR CONTRIBUTIONS

The authors have both made substantial contributions to conception and design, acquisition of data, analysis and interpretation of data; jointly wrote the manuscript; gave final approval of the version to be published; and have agreed to be accountable for all aspects of the work in ensuring that questions related to the accuracy or integrity of any part of the work are appropriately investigated and resolved. The first author significantly contributed to all five surveys described herein, and the second author significantly contributed to surveys from 2009 to 2019.

## ETHICS STATEMENT

Surveys in 2004 and 2005 were carried out as a workforce data gathering exercise for a national body (National Institute for Mental Health). Later surveys were registered as service evaluations with the R&D department of the NHS trust of Author 2, as the projects did not meet the criteria for research following the HRA guidelines (HRA, n.d.). The survey did not record personal identifiers, and individual trust's data were anonymised.

## Data Availability

The data that support the findings of this study are available from the corresponding author upon reasonable request.
